# Supplementary material for: Hydroxyzine Use and Mortality in Patients Hospitalized for COVID-19: A Multicenter Observational Study
Source: J Clin Med. 2021 Dec 15;10(24):5891. doi: 10.3390/jcm10245891 (PMC8707307; doi:10.3390/jcm10245891)
Supplement: Supplementary file 1 [file jcm-10-05891-s001.zip › jcm-1491621-supplementary.pdf]

SUPPLEMENTARY MATERIAL

Figure S1. Quantitative bias analysis based on the observed residual differences in age (A) and other patients characteristics (B) with an apparent OR of 0.51....................................... 2

Table S1. Associations of baseline clinical characteristics with mortality in the cohort of patients who had been admitted to the hospital for Covid-19 (N=15,103). 3

Table S2. Association between hydroxyzine use within the 3 months prior to hospital admission and not during the visit for COVID-19 versus patients with hydroxyzine use in the first 48h of hospitalization for COVID-19 and mortality (N=15,078)[a] 7

Table S3. Association between hydroxyzine use and mortality in the full sample and in the matched analytic sample, while adjusting for clinical severity of COVID-19 at baseline. 8

Table S4: Association between hydroxyzine use and mortality in the full sample and in the matched analytic sample separately among patients admitted in ICUs and those admitted in normal wards. 9

Table S5. Comparison of the mortality rate of patients who were prescribed hydroxyzine more than 48 hours after admission with those who received this medication within 48 hours from hospital admission and those who never received this treatment during the visit. 10

Table S6. Associations between hydroxyzine daily dose at baseline and mortality among patients receiving hydroxyzine (N=149). 11

Figure S1. Quantitative bias analysis based on the observed residual differences in age (A) and other patients characteristics (B) with an apparent OR of 0.51.

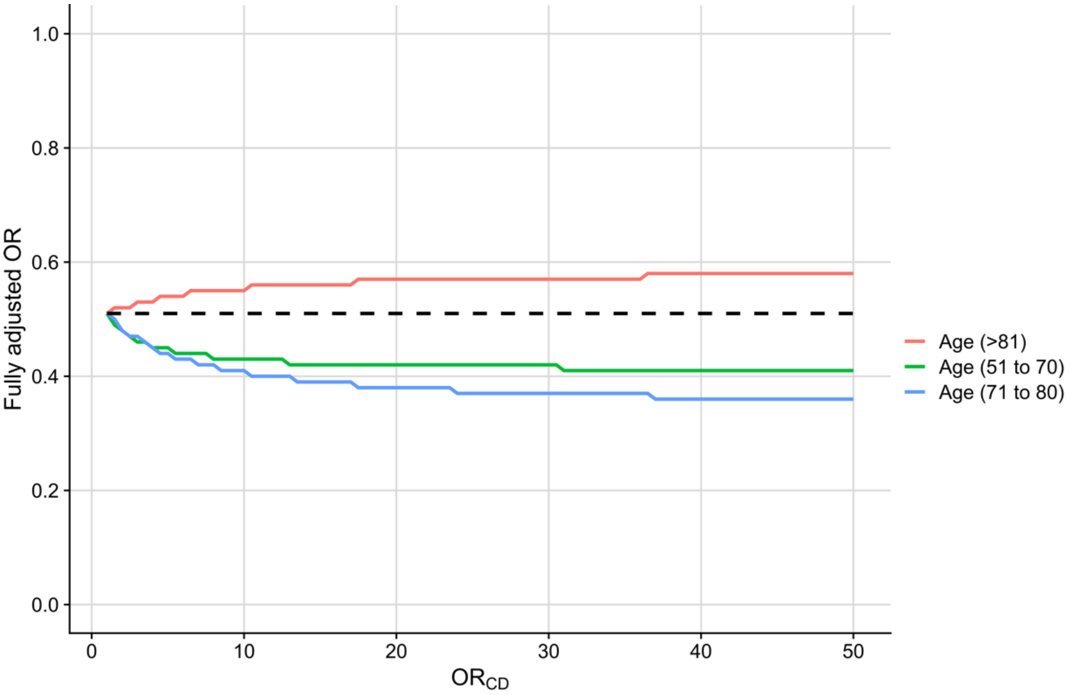

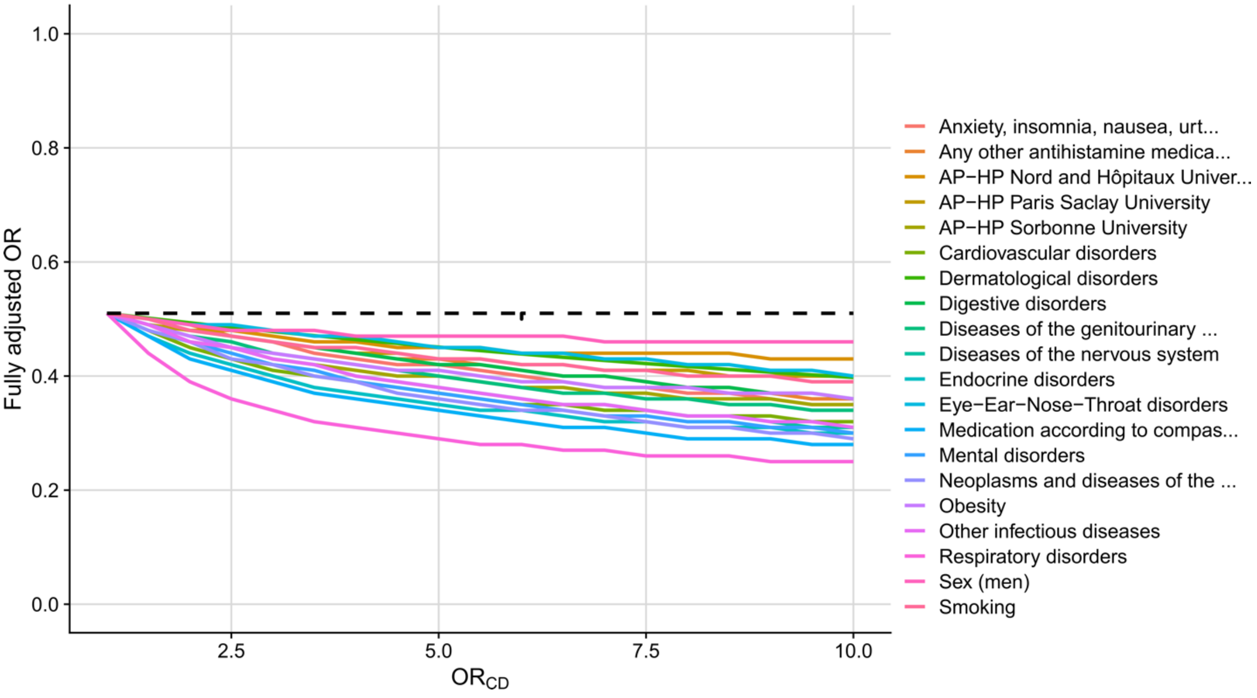

Table S1. Associations of baseline clinical characteristics with mortality in the cohort of patients who had been admitted to the hospital for Covid-19 (N=15,103).

				Endpoint of death		
	Full population (N=15,103)	With the end-point event (N=1,589)[+]	Without the end-point event (N=13,514)[+]	Crude analysis	Multivariable analysis	
	N (%)	N (%)	N (%)	HR (SE) / p-value	HR (SE) / p-value	Collinearity diagnosis (GVIF)
Patients' characteristics						
Age						1.21
18 to 50 years	5818 (38.5%)	54 (0.93%)	5764 (99.1%)	Ref.	Ref.	
51 to 70 years	4781 (31.7%)	370 (7.74%)	4411 (92.3%)	8.95 (6.71 - 11.94; <0.001*)	4.88 (3.63 - 6.56; <0.001*)	
71 to 80 years	1863 (12.3%)	395 (21.2%)	1468 (78.8%)	28.72 (21.49 - 38.39; <0.001*)	13.35 (9.89 - 18.02; <0.001*)	
More than 80 years	2641 (17.5%)	770 (29.2%)	1871 (70.8%)	43.93 (33.17 - 58.17; <0.001*)	29.94 (22.41 - 39.99; <0.001*)	
Sex						1.12
Women	7941 (52.6%)	598 (7.53%)	7343 (92.5%)	Ref.	Ref.	
Men	7162 (47.4%)	991 (13.8%)	6171 (86.2%)	1.97 (1.77 - 2.19; <0.001*)	1.72 (1.51 - 1.95; <0.001*)	
Hospital						1.12
AP-HP Centre - Paris University, Henri Mondor University Hospitals and at home hospitalization	7023 (46.5%)	510 (7.26%)	6513 (92.7%)	Ref.	Ref.	
AP-HP Nord and Hôpitaux Universitaires Paris Seine-Saint-Denis	4115 (27.2%)	525 (12.8%)	3590 (87.2%)	1.87 (1.64 - 2.12; <0.001*)	1.53 (1.32 - 1.78; <0.001*)	
AP-HP Paris Saclay University	1861 (12.3%)	291 (15.6%)	1570 (84.4%)	2.37 (2.03 - 2.76; <0.001*)	0.96 (0.80 - 1.16; 0.700)	
AP-HP Sorbonne University	2104 (13.9%)	263 (12.5%)	1841 (87.5%)	1.82 (1.56 - 2.14; <0.001*)	0.91 (0.76 - 1.09; 0.305)	
Obesity [a]						1.10
Yes	2047 (13.6%)	344 (16.8%)	1703 (83.2%)	1.92 (1.68 - 2.18; <0.001*)	1.47 (1.26 - 1.72; <0.001*)	
No	13056 (86.4%)	1245 (9.54%)	11811 (90.5%)	Ref.	Ref.	

Smoking [b]						1.04
Yes	1287 (8.52%)	234 (18.2%)	1053 (81.8%)	2.04 (1.75 - 2.38; <0.001*)	1.10 (0.92 - 1.32; 0.282)	
No	13816 (91.5%)	1355 (9.81%)	12461 (90.2%)	Ref.	Ref.	
Other medications						
Medication according to compassionate use or as part of a clinical trial [c]						1.14
Yes	1890 (12.5%)	341 (18.0%)	1549 (82.0%)	2.11 (1.85 - 2.41; <0.001*)	0.97 (0.83 - 1.13; 0.701)	
No	13213 (87.5%)	1248 (9.45%)	11965 (90.6%)	Ref.	Ref.	
Any other antihistamine medication						1.01
Yes	163 (1.08%)	21 (12.9%)	142 (87.1%)	1.26 (0.8 – 2.00; 0.324)	0.65 (0.39 - 1.08; 0.098)	
No	14940 (98.9%)	1568 (10.5%)	13372 (89.5%)	Ref.	Ref.	
Medical indications of hydroxyzine prescription						
Anxiety, insomnia, nausea, urticaria or pruritus						1.29
Yes	363 (2.40%)	131 (36.1%)	232 (63.9%)	5.14 (4.12 - 6.42; <0.001*)	1.15 (0.86 - 1.54; 0.333)	
No	14740 (97.6%)	1458 (9.89%)	13282 (90.1%)	Ref.	Ref.	
Medical comorbidities						
Other infectious diseases [d]						1.22
Yes	765 (5.07%)	222 (29.0%)	543 (71.0%)	3.88 (3.29 - 4.58; <0.001*)	1.14 (0.92 - 1.41; 0.232)	
No	14338 (94.9%)	1367 (9.53%)	12971 (90.5%)	Ref.	Ref.	
Neoplasms and diseases of the blood [e]						1.19
Yes	903 (5.98%)	273 (30.2%)	630 (69.8%)	4.24 (3.64 - 4.94; <0.001*)	1.24 (1.02 - 1.5; 0.030*)	
No	14200 (94.0%)	1316 (9.27%)	12884 (90.7%)	Ref.	Ref.	
Mental disorders [f]						1.19
Yes	852 (5.64%)	323 (37.9%)	529 (62.1%)	6.26 (5.39 - 7.28; <0.001*)	1.32 (1.08 - 1.6; 0.006*)	
No	14251 (94.4%)	1266 (8.88%)	12985 (91.1%)	Ref.	Ref.	

Diseases of the nervous system [g]						1.33
Yes	641 (4.24%)	233 (36.3%)	408 (63.7%)	5.52 (4.65 - 6.54; <0.001*)	1.17 (0.93 - 1.47; 0.172)	
No	14462 (95.8%)	1356 (9.38%)	13106 (90.6%)	Ref.	Ref.	
Cardiovascular disorders [h]						1.74
Yes	2166 (14.3%)	726 (33.5%)	1440 (66.5%)	7.05 (6.30 - 7.90; <0.001*)	1.31 (1.11 - 1.54; 0.001*)	
No	12937 (85.7%)	863 (6.67%)	12074 (93.3%)	Ref.	Ref.	
Respiratory disorders [i]						1.78
Yes	3549 (23.5%)	1004 (28.3%)	2545 (71.7%)	7.40 (6.62 - 8.26; <0.001*)	3.57 (3.04 - 4.20; <0.001*)	
No	11554 (76.5%)	585 (5.06%)	10969 (94.9%)	Ref.	Ref.	
Digestive disorders [j]						1.14
Yes	524 (3.47%)	146 (27.9%)	378 (72.1%)	3.52 (2.88 - 4.29; <0.001*)	0.91 (0.72 - 1.16; 0.464)	
No	14579 (96.5%)	1443 (9.90%)	13136 (90.1%)	Ref.	Ref.	
Dermatological disorders [k]						1.07
Yes	155 (1.03%)	63 (40.6%)	92 (59.4%)	6.02 (4.35 - 8.33; <0.001*)	1.54 (1.05 - 2.26; 0.027*)	
No	14948 (99.0%)	1526 (10.2%)	13422 (89.8%)	Ref.	Ref.	
Diseases of the musculoskeletal system [l]						1.12
Yes	375 (2.48%)	109 (29.1%)	266 (70.9%)	3.67 (2.92 - 4.61; <0.001*)	0.77 (0.59 - 1.02; 0.069)	
No	14728 (97.5%)	1480 (10.0%)	13248 (90.0%)	Ref.	Ref.	
Diseases of the genitourinary system [m]						1.29
Yes	903 (5.98%)	390 (43.2%)	513 (56.8%)	8.24 (7.14 - 9.52; <0.001*)	2.31 (1.92 - 2.78; <0.001*)	
No	14200 (94.0%)	1199 (8.44%)	13001 (91.6%)	Ref.	Ref.	
Endocrine disorders [n]						1.78
Yes	2201 (14.6%)	650 (29.5%)	1551 (70.5%)	5.34 (4.77 - 5.98; <0.001*)	1.01 (0.85 - 1.19; 0.932)	
No	12902 (85.4%)	939 (7.28%)	11963 (92.7%)	Ref.	Ref.	
Eye-Ear-Nose-Throat disorders [o]						1.07
Yes	167 (1.11%)	50 (29.9%)	117 (70.1%)	3.72 (2.66 - 5.2; <0.001*)	0.67 (0.45 - 0.99; 0.047*)	
No	14936 (98.9%)	1539 (10.3%)	13397 (89.7%)	Ref.	Ref.	

[a] Defined as having a body-mass index higher than 30 kg/m^2 or an International Statistical Classification of Diseases and Related Health Problems (ICD-10) diagnosis code for obesity (E66.0, E66.1, E66.2, E66.8, E66.9).
[b] Current Smoking status was self-reported.
[c] Any medication prescribed as part of a clinical trial or according to compassionate use (e.g., hydroxychloroquine, azithromycin, remdesivir, tocilizumab, sarilumab or dexamethasone).
[d] Assessed using ICD-10 diagnosis codes for certain infectious and parasitic diseases (A00-B99).
[e] Assessed using ICD-10 diagnosis codes for neoplasms (C00-D49) and diseases of the blood and blood-forming organs, and certain disorders involving the immune mechanism (D50-D89).
[f] Assessed using ICD-10 diagnosis codes for mental, behavioural, and neurodevelopmental disorders (F01-F99).
[g] Assessed using ICD-10 diagnosis codes for diseases of the nervous system (G00-G99).
[h] Assessed using ICD-10 diagnosis codes for diseases of the circulatory system (I00-I99).
[i] Assessed using ICD-10 diagnosis codes for diseases of the respiratory system (J00-J99).
[j] Assessed using ICD-10 diagnosis codes for diseases of the digestive system (K00-K95).
[k] Assessed using ICD-10 diagnosis codes for diseases of the skin and subcutaneous tissue (L00-L99).
[l] Assessed using ICD-10 diagnosis codes for diseases of the musculoskeletal system, and connective tissue (M00-M99).
[m] Assessed using ICD-10 diagnosis codes for diseases of the genitourinary system (N00-N99).
[n] Assessed using ICD-10 diagnosis codes for endocrine, nutritional and metabolic diseases (E00-E89).
[o] Assessed using ICD-10 diagnosis codes for diseases of the eye and adnexa (H00-H59), and diseases of the ear and mastoid process (H60-H95).
[+] The percentages in this column are expressed by row.
* Two-sided p-value is significant ($p<0.05$).
Abbreviations: HR, hazard ratio; CI, confidence interval.

Table S2. Association between hydroxyzine use within the 3 months prior to hospital admission and not during the visit for COVID-19 versus patients with hydroxyzine use in the first 48h of hospitalization for COVID-19 and mortality (N=15,078)[a]

	Number of events / Number of patients	Crude logistic regression analysis	Multivariable logistic regression analysis [b]	Multivariable logistic regression analysis [c]
	N (%)	OR (95%CI; p-value)	AOR (95%CI; p-value)	AOR (95%CI; p-value)
Hydroxyzine use during the visit for COVID-19	13 / 139 (9.4%)	Ref.	Ref.	Ref.
Hydroxyzine only within the last 3 months prior to the visit	24 / 146 (16.4%)	1.91 (0.93 - 3.91; 0.079)	4.26 (1.92 - 9.46; <0.001*)	4.3 (1.92 - 9.64; <0.001*)
No hydroxyzine	1547 / 14793 (10.5%)	1.13 (0.64 - 2.01; 0.672)	2.40 (1.27 - 4.53; 0.007*)	2.54 (1.34 - 4.82; 0.004*)

[a] 25 patients with hydroxyzine both within the last 3 months and in the first 48 hours of hospitalization were excluded from this analysis.
[b] Adjusted by age; sex; anxiety, insomnia, nausea, urticaria or pruritus; other infectious diseases; neoplasms and diseases of the blood; mental disorders; diseases of the nervous system; cardiovascular disorders; respiratory disorders; digestive disorders; dermatological disorders; diseases of the musculoskeletal system; diseases of the genitourinary system; endocrine disorders; and eye-ear-nose-throat disorders.
[c] Adjusted by age; sex; hospital; obesity; smoking status; medication according to compassionate use or as part of a clinical trial; any other antihistamine medication; anxiety, insomnia, nausea, urticaria or pruritus; other infectious diseases; neoplasms and diseases of the blood; mental disorders; diseases of the nervous system; cardiovascular disorders; respiratory disorders; digestive disorders; dermatological disorders; diseases of the musculoskeletal system; diseases of the genitourinary system; endocrine disorders; and eye-ear-nose-throat disorders.
* p-value is significant (p<0.05).
Abbreviations: OR, odds ratio; AOR, adjusted odds ratio; CI, confidence interval.

Table S3. Association between hydroxyzine use and mortality in the full sample and in the matched analytic sample, while adjusting for clinical severity of COVID-19 at baseline.

	Number of events / Number of patients	Multivariable logistic regression analysis [a]
	N (%)	AOR (95%CI; p-value)
Hydroxyzine	18 / 164 (11.0%)	0.49 (0.28 - 0.86; 0.013*)
No hydroxyzine	1571 / 14939 (10.5%)	Ref.

[a] Adjusted by age; sex; hospital; obesity; smoking status; medication according to compassionate use or as part of a clinical trial; any other antihistamine medication; anxiety, insomnia, nausea, urticaria or pruritus; other infectious diseases; neoplasms and diseases of the blood; mental disorders; diseases of the nervous system; cardiovascular disorders; respiratory disorders; digestive disorders; dermatological disorders; diseases of the musculoskeletal system; diseases of the genitourinary system; endocrine disorders; eye-ear-nose-throat disorders; and clinical severity of COVID-19 at baseline.

* p-value is significant ($p<0.05$).

Abbreviations: OR, odds ratio; AOR, adjusted odds ratio; CI, confidence interval; NA, not applicable.

Table S4: Association between hydroxyzine use and mortality in the full sample and in the matched analytic sample separately among patients admitted in ICUs and those admitted in normal wards.

	Number of events / Number of patients	Crude logistic regression analysis	Multivariable logistic regression analysis [a]	Multivariable logistic regression analysis [b]	Number of events / Number of patients	Univariate logistic regression in the matched analytic sample (1:2)	Multivariable logistic regression analysis in the matched analytic sample (1:2)
	N (%)	OR (95%CI; p-value)	AOR (95%CI; p-value)	AOR (95%CI; p-value)	N (%)	OR (95%CI; p-value)	AOR (95%CI; p-value)
**With ICU (N=809)**							
Hydroxyzine	2 / 24 (8.3)	0.15 (0.04 - 0.66; 0.012*)	0.08 (0.02 – 0.37; 0.001*)	0.08 (0.02 – 0.38; 0.002*)	2 / 24 (8.3)	0.14 (0.03 – 0.66; 0.013*)	0.14 (0.02 – 0.79; 0.026*) [c]
No hydroxyzine	291 / 785 (37.1)	Ref.	Ref.	Ref.	19 / 48 (39.6)	Ref.	Ref.
**Without ICU (N=14,294)**							
Hydroxyzine	16 / 140 (11.4)	1.30 (0.77 - 2.19; 0.329)	0.73 (0.40 – 1.32; 0.293)	0.44 (0.20 – 0.98; 0.044*)	16 / 140 (11.4)	0.45 (0.25 – 0.82; 0.009*)	0.42 (0.23 – 0.78; 0.006*) [d]
No hydroxyzine	1280 / 14154 (9.0)	Ref.	Ref.	Ref.	62 / 280 (22.1)	Ref.	Ref.

[a] Adjusted by age; sex; anxiety, insomnia, nausea, urticaria or pruritus; other infectious diseases; neoplasms and diseases of the blood; mental disorders; diseases of the nervous system; cardiovascular disorders; respiratory disorders; digestive disorders; dermatological disorders; diseases of the musculoskeletal system; diseases of the genitourinary system; endocrine disorders; and eye-ear-nose-throat disorders.
[b] Adjusted by age; sex; hospital; obesity; smoking status; medication according to compassionate use or as part of a clinical trial; any other antihistamine medication; anxiety, insomnia, nausea, urticaria or pruritus; other infectious diseases; neoplasms and diseases of the blood; mental disorders; diseases of the nervous system; cardiovascular disorders; respiratory disorders; digestive disorders; dermatological disorders; diseases of the musculoskeletal system; diseases of the genitourinary system; endocrine disorders; and eye-ear-nose-throat disorders.
[c] Adjusted by age; hospital, smoking status; mental disorders; diseases of the nervous system; cardiovascular disorders; and digestive disorders.
[d] Adjusted by age; sex; obesity; diseases of the musculoskeletal system; endocrine disorders; and and eye-ear-nose-throat disorders.
* p-value is significant ($p<0.05$).
Abbreviations: OR, odds ratio; AOR, adjusted odds ratio; CI, confidence interval.

Table S5. Comparison of the mortality rate of patients who were prescribed hydroxyzine more than 48 hours after admission with those who received this medication within 48 hours from hospital admission and those who never received this treatment during the visit.

	Number of events / Number of patients	Crude logistic regression analysis	Multivariable logistic regression analysis[a]	Multivariable logistic regression analysis [b]	Number of events / Number of patients	Univariate logistic regression in the matched analytic sample (1:2)	Multivariable logistic regression analysis in the matched analytic sample (1:2)
	N (%)	OR (95%CI; p-value)	AOR (95%CI; p-value)	AOR (95%CI; p-value)	N (%)	OR (95%CI; p-value)	AOR (95%CI; p-value)
Hydroxyzine after more than 48 hours from hospital admission	13 / 65 (20.0%)	2.03 (0.93 - 4.43; 0.076)	3.87 (1.37 - 10.89; 0.010*)	4.78 (1.40 - 16.33; 0.013*)	13 / 65 (20.0%)	3.00 (1.24 - 7.28; 0.015*)	4.53 (1.39 - 14.77; 0.012*)[c]
Hydroxyzine within 48 hours of hospital admission	18 / 164 (11.0%)	Ref.	Ref.	Ref.	10 / 130 (7.7%)	Ref.	Ref.
Hydroxyzine after more than 48 hours from hospital admission	13 / 65 (20.0%)	2.13 (1.16 - 3.92; 0.015*)	1.31 (0.67 - 2.59; 0.430)	1.37 (0.69 - 2.70; 0.366)	13 / 65 (20.0%)	1.30 (0.60 - 2.79; 0.505)	1.58 (0.68 - 3.63; 0.286)[d]
No hydroxyzine during the hospitalization	1571 / 14939 (10.5%)	Ref.	Ref.	Ref.	21 / 130 (16.2%)	Ref.	Ref.
Hydroxyzine within 48 hours of hospital admission	18 / 164 (11.0%)	1.05 (0.64 - 1.72; 0.849)	0.53 (0.31 - 0.91; 0.023*)	0.51 (0.29 - 0.88; 0.016*)	18 / 164 (11.0%)	0.45 (0.26 - 0.78; 0.004*)	0.43 (0.24 - 0.76; 0.004*)[e]
No hydroxyzine during the hospitalization	1571 / 14939 (10.5%)	Ref.	Ref.	Ref.	71 / 328 (21.6%)	Ref.	Ref.

[a] Adjusted by age; sex; anxiety, insomnia, nausea, urticaria or pruritus; other infectious diseases; neoplasms and diseases of the blood; mental disorders; diseases of the nervous system; cardiovascular disorders; respiratory disorders; digestive disorders; dermatological disorders; diseases of the musculoskeletal system; diseases of the genitourinary system; endocrine disorders; and eye-ear-nose-throat disorders.

[b] Adjusted by age; sex; hospital; obesity; smoking status; medication according to compassionate use or as part of a clinical trial; any other antihistamine medication; anxiety, insomnia, nausea, urticaria or pruritus; other infectious diseases; neoplasms and diseases of the blood; mental disorders; diseases of the nervous system; cardiovascular disorders; respiratory disorders; digestive disorders; dermatological disorders; diseases of the musculoskeletal system; diseases of the genitourinary system; endocrine disorders; and eye-ear-nose-throat disorders.

[c] Adjusted by age; sex; hospital; medication according to compassionate use or as part of a clinical trial; anxiety, insomnia, nausea, urticaria or pruritus; neoplasms and diseases of the blood; mental disorders; diseases of the nervous system; cardiovascular disorders; respiratory disorders, diseases of the genitourinary system; endocrine disorders; and eye-ear-nose-throat disorders.

[d] Adjusted by age; smoking status; mental disorders; and eye-ear-nose-throat disorders.

[e] Adjusted by age; hospital; and obesity.

* p-value is significant ($p<0.05$).

Abbreviations: OR, odds ratio; AOR, adjusted odds ratio; CI, confidence interval.

Table S6. Associations between hydroxyzine daily dose at baseline and mortality among patients receiving hydroxyzine (N=149).

	Number of events / Number of patients	Crude logistic regression analysis	Multivariable logistic regression analysis [a]	Multivariable logistic regression analysis [b]
	N (%)	OR (95%CI; p-value)	AOR (95%CI; p-value)	AOR (95%CI; p-value)
Higher doses	15 / 137 (10.9%)	0.61 (0.12 - 3.08; 0.554)	0.36 (0.03 - 4.28; 0.418)	NA
Lower doses	2 / 12 (16.7%)	Ref.	Ref.	Ref.

Because daily dose could not be ascertained with certainty in 15 (9.1%) patients, these patients have been excluded from this analysis.
The variable 'daily dose' has been dichotomized by the median (i.e. 25 mg).
[a] Adjusted by age; sex; anxiety, insomnia, nausea, urticaria or pruritus; other infectious diseases; neoplasms and diseases of the blood; mental disorders; diseases of the nervous system; cardiovascular disorders; respiratory disorders; digestive disorders; dermatological disorders; diseases of the musculoskeletal system; diseases of the genitourinary system; endocrine disorders; and eye-ear-nose-throat disorders.
[b] Adjusted by age; sex; hospital; obesity; smoking status; medication according to compassionate use or as part of a clinical trial; any other antihistamine medication; anxiety, insomnia, nausea, urticaria or pruritus; other infectious diseases; neoplasms and diseases of the blood; mental disorders; diseases of the nervous system; cardiovascular disorders; respiratory disorders; digestive disorders; dermatological disorders; diseases of the musculoskeletal system; diseases of the genitourinary system; endocrine disorders; and eye-ear-nose-throat disorders.
Abbreviations: OR, odds ratio; AOR, adjusted odds ratio; CI, confidence interval; NA, not applicable.
